# Prognostic and Predictive Value of m6A “Eraser” Related Gene Signature in Gastric Cancer

**DOI:** 10.3389/fonc.2021.631803

**Published:** 2021-02-26

**Authors:** Xin Xu, En Zhou, Jun Zheng, Chihao Zhang, Yinghua Zou, Jiayun Lin, Jiwei Yu

**Affiliations:** ^1^Department of General Surgery, Shanghai Ninth People’s Hospital, School of Medicine, Shanghai Jiao Tong University, Shanghai, China; ^2^Department of Cardiology, Shanghai Ninth People’s Hospital, School of Medicine, Shanghai Jiao Tong University, Shanghai, China; ^3^Department of Anesthesiology, Shuguang Hospital Affiliated to Shanghai University of Traditional Chinese Medicine, Shanghai, China

**Keywords:** gastric cancer, FTO, ALKBH5, prognosis, immune infiltrating cells

## Abstract

**Background:**

N6-methyladenosine (m6A) RNA modification plays a critical role in gastric cancer (GC). However, the relationship between the m6A “eraser”, FTO, and ALKBH5, and the prognosis of GC still remains unclear. This study aimed to evaluate the effect of FTO and ALKBH5 on the prognosis of patients and their potential roles in GC.

**Materials and Methods:**

A total of 738 GC samples with clinical information obtained from two independent datasets were included and divided into training set and testing set. Differential expression analysis of the m6A “eraser” related genes was performed. The LASSO Cox regression model was constructed to analyze the m6A “eraser” related risk genes. The univariate and multivariate Cox regression model were employed to identify the independent prognostic factors. Kaplan-Meier method was used for survival analysis. A nomogram model was then carried out to predict the prognosis of GC patients. Additionally, GO and KEGG analyses were conducted to identify the potential role of the m6A “eraser” related genes in GC. The relative proportion of 22 different genotypes in immune infiltrating cells was calculated by CIBERSORT algorithm.

**Results:**

In total, nine m6A “eraser” related risk genes and risk scores were obtained and calculated. Patients in high-risk group demonstrated significantly worse prognosis than those in low-risk group. Age, stage, and risk score were considered as independent prognostic factors. The nomogram model constructed accurately predicted the 3-year and 5-year overall survival (OS) of patients. Furthermore, m6A “eraser” might play a functional role in GC. The expression of m6A “eraser” leads to changes in tumor immune microenvironment.

**Conclusions:**

FTO and ALKBH5 showed association with the prognosis of GC. The m6A “eraser” related genes, which is considered as a reliable prognostic and predictive tool, assists in predicting the OS in GC patients.

## Introduction

Gastric cancer (GC) is one of the most common malignancies (1), and is the second most common cause for cancer-related deaths worldwide. What is more, it is the most prevalent type of cancer in Eastern Asia (2), causing a huge burden to patients (3). Therefore, it is necessary to identify the potential therapeutic targets for GC and improve the prognosis of patients.

N6-methyladenosine (m6A) RNA modification plays an important role in RNA splicing, export, stability, and translation, and is considered as the most abundant epigenetic methylation modification of mRNAs and non-coding RNAs (ncRNAs) (4, 5). According to the previous studies, the process of m6A modification regulated by m6A regulators is invertible and dynamic, which includes “writers” (methyltransferases), “eraser” (demethylases), and “readers” (m6A binding proteins) (6, 7). So far, only the fat mass and obesity-associated (FTO) and ALKBH5 (α-ketoglutarate-dependent dioxygenase alkB homologue 5) have been found to act as “erasers” for m6A demethylation. ALKBH5 is a homologue of FTO, and their existence ensures the balance of m6A modification in the transcriptome (8).

The m6A “erasers” act as demethylase by using ferrum as a co-factor and α-ketoglutaric acid as co-matrix to remove m6A (9), which appear to have limited functions under normal physiological conditions (6), but might induce the tumorigenesis of GC. Previous studies have shown that ALKBH5 was able to promote the proliferation of glioblastoma stem-like cells by regulating the expression of FOXM1 (10). Moreover, FTO could promote tumor progression by regulating multiple signaling pathways in a variety of tumors (11, 12). Also, a recent bioinformatic research revealed FTO as an independent prognostic biomarker and a predictor of clinicopathological characteristics of GC (13). However, the specific mechanism of m6A “erasers”, FTO and ALKBH5, in the progression of GC still remains unclear.

In this study, 305 GC samples obtained from the Cancer Genome Atlas (TCGA) datasets and 433 GC samples obtained from the Gene Expression Omnibus (GEO) datasets were analyzed. Nine m6A “eraser” related risk genes were screened using a least absolute shrinkage and selection operator (LASSO) Cox regression model and a nomogram model was constructed to predict the overall survival (OS) of patients with GC. The prognostic and predictive accuracies of the classifier in the training set (TCGA datasets) were assessed and validated in the testing group (GSE84437 datasets). The prognostic and predictive efficacy of clinicopathological risk factors were also compared in this study. In addition, the potential role of m6A “eraser” in GC and its possible signaling pathways were explored, and the expression of m6A “eraser” showed certain effects on tumor immune cell infiltration.

## Materials and Methods

### Data Source and Acquisition

A total of 738 GC samples with clinical information obtained from TCGA (https://portal.gdc.cancer.gov), and GEO (https://www.ncbi.nlm.nih.gov/geo/) datasets were explored. The clinical information from TCGA was downloaded from the UCSC Xena database (http://xena.ucsc.edu), and the samples without clinical information were filtered out. The dataset from GEO (GSE84437) was processed using the chip platform (Illumina HumanHT-12 V3.0 expression beadchip, San Diego, CA, USA), which is the most commonly used in transcriptome analysis.

### Data Processing

For microarray data (GSE84437), the raw probe level data in each CEL file was processed by robust multi-array average (RMA) algorithm of the Affy package (14). For genes that match multiple probes, average probe values were used as the expression value (15). The TCGA dataset was normalized by log-transformation of the Fragment Per Kilobase Per Million Reads (FPKM) +1. The missing data in these two gene expression matrices were supplemented by the k-Nearest Neighbor (KNN) approach (k = 10) (16). The gene expression data were transformed by Z-score to avoid systematic errors in different experiments.

### Bioinformatic Analysis

The “edgeR” package was used to explore differential genes between high and low expression groups of m6A “eraser” FTO/ALKBH5, and the common differential expression genes were obtained using the “venn” package. The relationship between m6A “eraser”-related genes and the patient OS was evaluated with univariate Cox regression analysis by R software. Thereafter, the “glmnet” package was used to conduct LASSO Cox regression model (with the penalty parameter estimated by 10-fold cross-validation) (17). A risk score formula based on the expression levels of 9 m6A “eraser”-related genes for OS prediction was created, where the risk score was (3.74863 * expression level of NRP2) + (2.223197 * expression level of PARVA) + (0.626251 * expression level of LAMA4) + (0.463306 * expression level of EHD2) + (0.455573 * expression level of ANTXR2) + (0.074203 * expression level of DPYSL3) – (2.37796 * expression level of SDC3) – (2.47335 * MTMR12) – (2.71575 * expression level of SH3PXD2A). The risk scores were determined for all patients included in this study, and the median value was chosen as the cut-off value to divide patients into high- and low-risk groups.

To explore the potential function and signaling pathway related to m6A “eraser” in GC patients, Gene Ontology (GO) and the Kyoto Encyclopedia of Genes and Genomes (KEGG) pathways enrichment analyses were performed in m6A “eraser”-related differentially expressed genes using the “clusterProfiler” package. Meanwhile, by using the GC gene expression data, the relative proportion of 22 different genotypes in immune infiltrating cells was calculated by CIBERSORT algorithm.

### Statistical Analysis

SPSS version 23.0 (SPSS Inc., Chicago, IL, USA) and R software for windows version and R-4.0.2 (The R Foundation for Statistical Computing, Vienna, Austria) were used for data analysis. Continuous variables were compared using the t-test and categorical variables were compared using the χ2 test in both the groups. Univariate and multivariate Cox regression models were chosen to identify independent prognostic factors for OS. Hazard ratios (HRs) and 95% confidence intervals (95% CIs) were also determined. Kaplan-Meier method was used to analyze the association between variables and OS, and log-rank test was used for comparing the survival curves. A nomogram model as generated by a Cox regression model which was used in univariate and multivariate analyses. The “rms” package was used to generate nomogram and calibration plots. Decision curve analysis (DCA) was applied to identify the clinical feasibility of the nomogram (18). A P-value of <0.05 was considered to be statistically significant.

## Results

### Identification of the Nine m6A “Eraser” Related Genes

**Figure 1:**
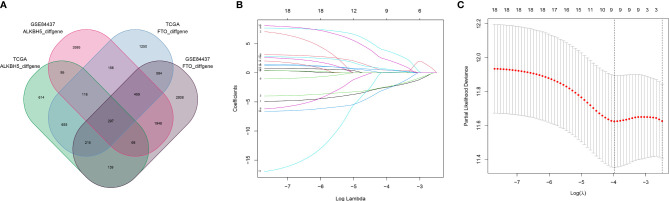
Screening of 9 m6A “eraser” related risk genes. **(A)** Venn diagram showing 297 commonly and differentially expressed genes in four groups with different data. **(B, C)** LASSO regression was employed to calculate the minimum criteria **(B)** and coefficients **(C)**.

A total of 305 GC samples from the TCGA dataset were assigned to the training set, and 433 GC samples from the GSE84437 were assigned to the testing set. According to the expression levels of m6A “eraser,” FTO and ALKBH5, both training and testing sets were divided into FTO high and low expression groups and ALKBH5 high and low groups. The baseline demographic characteristics were shown in **Table 1**. A differential gene expression analysis was performed in FTO/ALKBH5 high and low groups from the training and testing sets (**Supplementary Figures 1A–D**). Two hundred ninety-seven common differentially expressed genes were identified in these four groups (**Figure 1A**), and 18 genes with p-values less than 0.05 were identified after univariate Cox regression analysis. After that, a Lasso Cox model was built to screen the risk genes to analyze the OS of GC patients, which generated nine m6A “eraser” related risk genes as well as the coefficients of each gene (**Figures 1B, C**, and **Supplementary Table 1**).

**Table 1:** Baseline clinicopathological characteristics.

Variables	FTO Low (N = 152)	FTO High (N = 153)	P value	ALKBH5 Low (N = 152)	ALKBH5 High (N = 153)	P value
**Age, n (%)**			**0.045**			0.871
≤60	108 (71.1%)	91 (59.5%)		98 (64.5%)	101 (66.0%)	
>60	44 (28.9%)	62 (40.5%)		54 (35.5%)	52 (34.0%)	
**Gender, n (%)**			0.667			0.254
female	54 (35.5%)	59 (38.6%)		51 (33.6%)	62 (40.5%)	
male	98 (64.5%)	94 (61.4%)		101 (66.4%)	91 (59.5%)	
**Stage, n (%)**			0.087			0.339
Stage I	25 (16.4%)	14 (9.15%)		20 (13.2%)	19 (12.4%)	
Stage II	54 (35.5%)	45 (29.4%)		51 (33.6%)	48 (31.4%)	
Stage III	59 (38.8%)	76 (49.7%)		61 (40.1%)	74 (48.4%)	
Stage IV	14 (9.21%)	18 (11.8%)		20 (13.2%)	12 (7.84%)	
**Grade, n (%)**			**<0.001**			1
G1-2	71 (46.7%)	41 (26.8%)		56 (36.8%)	56 (36.6%)	
G3-4	81 (53.3%)	112 (73.2%)		96 (63.2%)	97 (63.4%)	
**T, n (%)**			**0.015**			0.974
T1-2	47 (30.9%)	28 (18.3%)		38 (25.0%)	37 (24.2%)	
T3-4	105 (69.1%)	125 (81.7%)		114 (75.0%)	116 (75.8%)	
**M, n (%)**			0.254			0.478
M0	145 (95.4%)	140 (91.5%)		140 (92.1%)	145 (94.8%)	
M1	7 (4.61%)	13 (8.50%)		12 (7.89%)	8 (5.23%)	
**N, n (%)**			0.592			1
N0	103 (67.8%)	109 (71.2%)		106 (69.7%)	106 (69.3%)	
N+	49 (32.2%)	44 (28.8%)		46 (30.3%)	47 (30.7%)	
**Subtype, n (%)**			0.062			**0.041**
MSS	92 (60.5%)	112 (73.2%)		104 (68.4%)	100 (65.4%)	
MSI-L	28 (18.4%)	20 (13.1%)		29 (19.1%)	19 (12.4%)	
MSI-H	32 (21.1%)	21 (13.7%)		19 (12.5%)	34 (22.2%)	

The bold value in Table 1 means that the p value is less than 0.05, and the difference is statistically significant.

### Identification and Validation of Survival Prediction Ability of the Nine m6A “Eraser” Related Risk Genes

**Figure 2:**
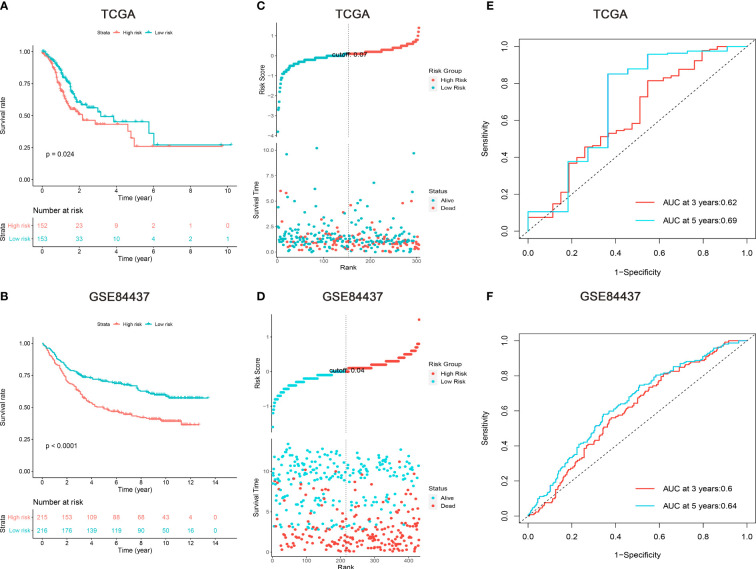
Survival analysis of gastric cancer patients in the TCGA and GSE84437 cohorts. **(A, B)** Kaplan-Meier survival analysis, **(C, D)** risk score by the nine m6A “eraser” relater risk genes, patients’ survival status and time, **(E, F)** time-dependent ROC curves. The AUCs of 3 and 5 years were used to assess prognostic accuracy and log-rank test was used to calculated the p-value.

**Figure 3:**
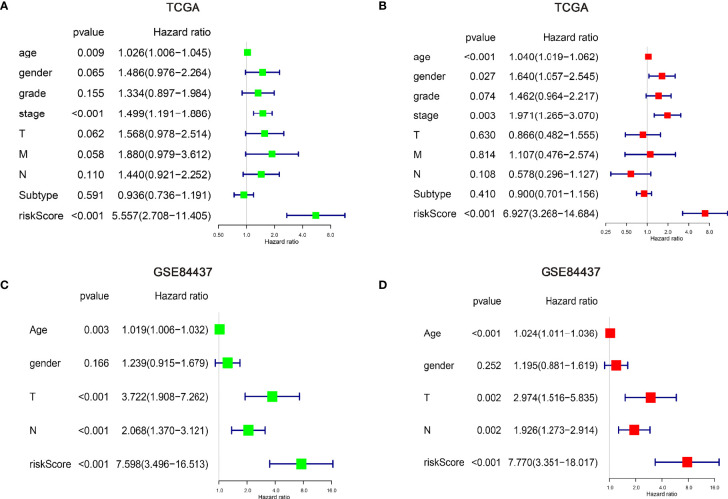
Explore the independent prognostic risk factors in gastric cancer patients. Univariate and multivariate Cox regression analysis of clinicopathological characteristics were performed in the TCGA **(A, B)** and GSE84437 **(C, D)** cohorts. And the hazard ratios (HR) and 95% confidence intervals (CI) were calculated, respectively.

According to Lasso Cox model, patients in the training set were divided into low- and high-risk subgroups based on the median values of risk scores. Kaplan-Meier survival analysis revealed that patients in the high-risk subgroup had significantly worse prognosis than those in the low-risk subgroup (**Figures 2A, B**). The distributions of risk scores, survival time, and status were assessed, which indicated that patients with lower risk scores had better survival time (**Figures 2C, D**). The time-dependent ROC curves demonstrated that the nine m6A “eraser”-related risk genes had a promising ability to predict the OS of GC patients (**Figures 2E, F**). After multivariable adjustment by clinicopathological factors, the risk scores of nine m6A “eraser” related genes remained to be a powerful and independent factor (**Figures 3B, D**). All the above analyses showed similar results in both training and testing sets.

### Identification and Validation of Independent Factors for m6A “Eraser”

The relationship between the nine m6A “eraser”-related risk genes and clinicopathological factors was explored. The results revealed that DPYSL3, EHD2, PARVA, NRP2, ANTXR2, and LAMA4 as risky genes, and the high expression level of these genes predicted worse prognosis. In contrast, MTMR12, SDC3, and SH3PXD2A were found to be as protective genes, and the high expression level of these three genes predicted better prognosis (**Supplementary Figure 2A**).

Next, univariate and multivariate Cox regression analyses of clinicopathological factors were performed. The results showed age, stage, and risk score as independent risk factors in the training set. Meanwhile, age, T stage, N stage, and risk score were shown to be as independent risk factors in the testing set (**Figures 3A–D**). Although some differences existed between the training and testing sets, these differences indicated similar results as tumor staging was defined by T, N, and M stage. In addition, we further performed Cox regression analyses for each clinicopathological subgroup of GC patients (**Supplementary Figures 2B, C**), however, we did not find significant intra group differences.

### Risk of Clinicopathological Subgroup Analyses

**Figure 4:**
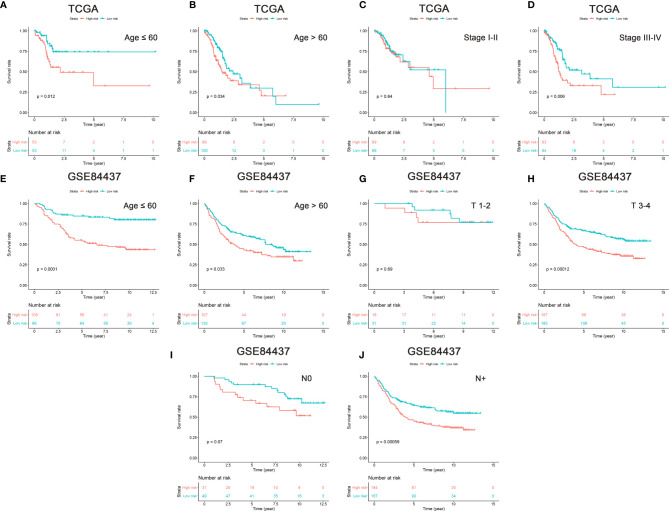
Prognostic analysis in the subtypes with each independent risk factor group. The age and stage subtypes were analyzed in TCGA cohort **(A–D)**, and the age, T and N were analyzed in GSE84437 cohort **(E–J)**. P-value was calculated by log-rank test.

Furthermore, survival analyses were performed for each independent risk factor of clinicopathological subgroup in GC patients. The results showed that high risk scores demonstrated worse OS in patients aged ≤60, >60, and stage III-IV subgroups in the TCGA cohort, and had significantly decreased OS in patients aged ≤60, >60, T3-4, and positive lymph node metastasis subgroups in GSE84437 cohort (**Figures 4A–J**). These data indicated that the nine m6A “eraser”-related genes could act as a potential predictor in GC patients.

### Construction and Validation of Nomogram Model

**Figure 5:**
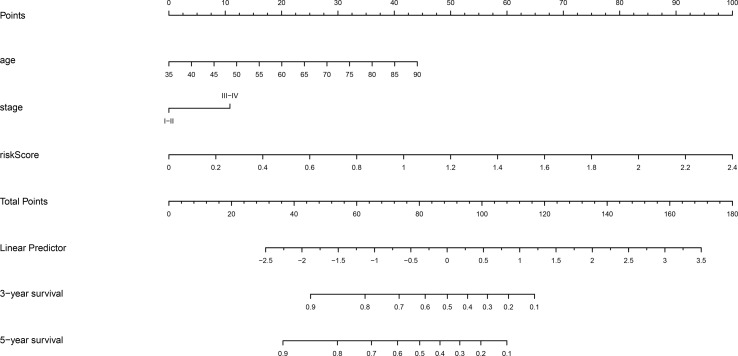
Nomogram prediction of 3-year and 5-year OS probability in gastric cancer. The nomogram model was constructed in the TCGA dataset, with age, stage, and risk score of nine m6A “eraser” related risk genes.

To create a clinically quantitative tool for predicting the OS in GC patients, a nomogram model using age, stage, and risk scores (based on the nine m6A “eraser”-related genes) was established in the TCGA dataset, and tested in the GSE84437 dataset (**Figure 5**). The calibration curves showed perfect observed and predicted ratios of 3-year and 5-year OS in the TCGA cohort (**Supplementary Figures 3A, B**). The nomogram DCA curves showed that if the threshold probability of 3-year and 5-year OS was more than 0.11 and 0.22 respectively, the use of nomogram could offer a higher net benefit than treating all patients or treating no patients (**Supplementary Figures 3C, D**). These data suggest that the nomogram model had a strong ability and accuracy in predicting the OS in GC patients.

### The Potential Role of m6A “Eraser” in Gastric Cancer

**Figure 6:**
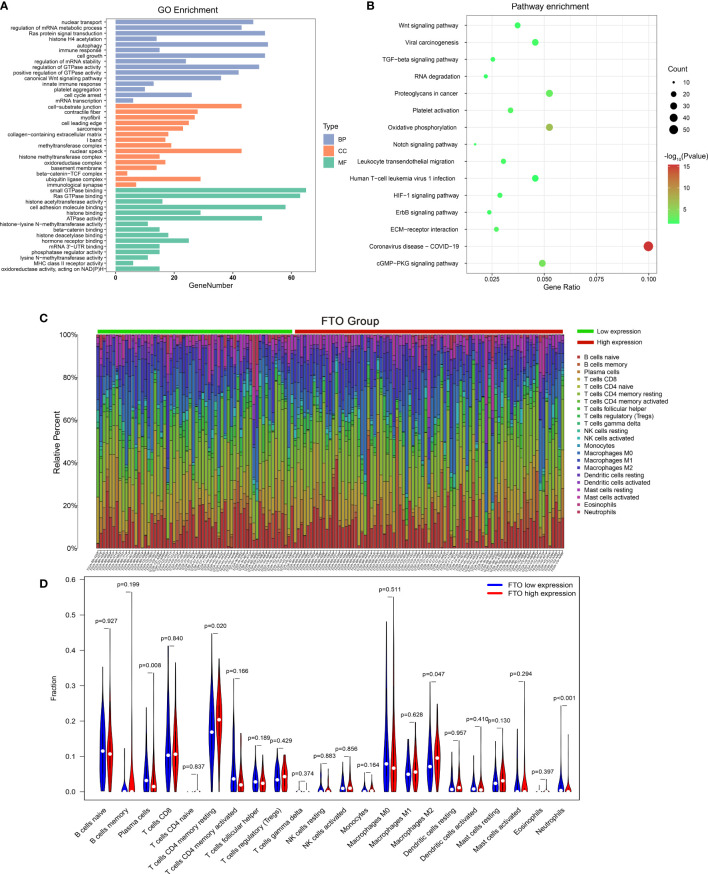
The potential role and function of m6A “eraser,” and the effect of FTO expression level on gastric cancer immune cells. The functional annotation of differential expression of m6A “eraser” related genes using GO **(A)** and KEGG pathway **(B)** analysis. **(C)** The proportion of immune cell subsets in the FTO high expression group and low expression group were analyzed in TCGA dataset. **(D)** The violin map showed statistical differences between the immune cells of different FTO expression groups.

To explore the potential roles of m6A “eraser” in GC, the GO and KEGG analyses were performed based on 297 common differentially expressed genes. The GO analyses showed that the functions of m6A “eraser” mainly focused on regulating tumor malignant biological behavior, including regulation of mRNA metabolic process, autophagy, cell growth, cell cycle arrest, platelet aggregation, and so on (**Figure 6A**), and the enriched items listed have played an important role in the progression of cancer. The KEGG analyses were primarily enriched in several cancer-associated pathways, such as Wnt signaling pathway, TGF-β signaling pathway, ECM-receptor interaction, and cGMP-PKG signaling pathway (**Figure 6B**).

### Correlation Between m6A “Eraser” and Immune Cells

Interestingly, in go enrichment analysis, we found that the function of m6A eraser is not only related to tumor progression, but also related to some human immune functions, such as, immune response, innate immune response, MHC class II receptor activity (**Figure 6A**). Therefore, we were curious to know whether the expression of the m6A “eraser” in GC was associated with immune cell infiltration, relevant studies based on TCGA datasets were conducted. Finally, among the 22 immune cell genotypes found, the high expression of FTO showed a positive correlation with the expression of T cells CD4 memory resting and macrophages M2, and showed a negative correlation with the expression of neutrophils (**Figures 6C, D**). The high expression of ALKBH5 was positively correlated with the expression of T cells CD4 memory resting and negatively correlated with that of dendritic cells resting and neutrophils (**Supplementary Figures 4A, B**). These results indicated that the immune microenvironment of tumor cells changes with changing expressions of FTO and ALKBH5 in GC patients.

## Discussion

In this study, a new prognostic tool based on the expression profile of 9 m6A “eraser” related risk genes has been established to predict the OS in GC patients. We discovered that the patients in high-risk group had a significantly decreased OS when compared to those in low-risk group. Multivariate Cox regression analysis revealed that the nine m6A “eraser” related risk genes were seemed to be independent risk factors for predicting OS in GC patients. Then, a nomogram model for GC based on m6A “eraser” related risk genes and other independent risk clinical characteristics was built to accurately predict 3- and 5-year OS. Functional enrichment analysis suggested that two m6A “eraser” genes were involved in the occurrence and progression of GC and could play a role by activating the classical tumor signaling pathways. In addition, FTO and ALKBH5 could also alter the immune microenvironment of GC tumor cells.

As GC is the second most common cause of cancer-related deaths around the world, the treatment of GC in every single patient results in different outcome. Thus, it is critical to find a prognostic factor for GC. Previous studies have reported that low-m6A signatures might predict adverse clinicopathological characteristics of GC (19). The m6A “eraser” provided a survival benefit in patients who were classified into the low-risk group. The use of m6A “eraser” based risk gene combinations assists in better identifying patient outcomes, which might in turn provide more powerful support for the development of treatment plans.

In addition, nine m6A “eraser” related risk genes screened from high-dimensional gene expression data by LASSO-Cox provides high prediction accuracy for prognosis and low correlation between the data, which can prevent overfitting, while some prediction tools select genes without the dimension of reduction, which might lead to overfitting (20, 21).

In previous studies, the m6A modification has been proved to play a critical role in GC (7, 13, 22, 23). However, most studies focused on the effect of m6A methylated modification rather than m6A demethylase (eraser) modification on GC. Yunshu Su et al. have found that m6A RNA methylation regulators (FTO, ALKBH5) act as independent prognostic markers and predictors of GC (13). Although it has been reported that the expression levels of FTO and ALKBH5 might affect the prognosis of GC patients, how to obtain potential prognosis of GC patients through the expression of m6A “eraser” and its potential role in GC still remains unknown.

In our study, the survival rates of several clinicopathological characteristics were analyzed. The results showed that the high-risk group had a significantly worse prognosis at any age, and the survival rate of patients with stages III and IV and positive lymph node metastasis was lower than that of the low-risk group. These results indicated that the expression of m6A “eraser” related genes assisted in predicting the outcome of GC more accurately.

Furthermore, the potential role of m6A “eraser” in GC *via* GO and KEGG pathways enrichment analyses was explored. These two analyses obviously revealed that m6A “eraser” is robustly associated with Wnt signaling pathway. Previous study has reported that the Wnt/β-catenin signaling pathway induced epithelial-mesenchymal transition to maintain the integrity of epithelial cells and tight/adherent junctions (24). Meanwhile, overactivation of canonical Wnt pathway has been determined in 30–50% of GC tissues and cell lines (25, 26). Combined with our findings, these results suggested that the m6A “eraser” related genes might affect the GC by regulating the function of cell adhesion *via* Wnt signaling pathway.

The immune system has been involved in the process of GC. Several studies have determined the characterization of immune microenvironment in GC. Some immune cells, like M2 macrophages, mast cells, monocytes, release spectrum of proinflammatory, angiogenic, lymphangiogenic, and immunomodulatory mediators were shown to play a pro-tumorigenic role (27). At the same time, other immune cells, including M1 macrophages, cytotoxic CD8+ T cells, and NK cells also play an anti-tumorigenic role in GC (27). Our study further explored the relationship between m6A “eraser” and immune cells. The results showed that both high expression FTO and ALKBH5 showed a positive correlation with the number of T cells CD4 memory resting and a negative correlation with the number of neutrophils. Recent studies have reported that resting memory CD4 T cells are closely related with the pathogenesis of various malignant tumors, including GC, and pointed out that they might be affected by the key genes (28). Numerous studies have demonstrated that neutrophils play a key role in various tumors, including the promotion of lymph node metastasis in early GC, and high levels of tumor-associated neutrophils also promote GC progression and are associated with poor clinical prognosis (29). These results suggest that m6A “eraser” might act as a key gene in changing the immune microenvironment by regulating the levels of T cells CD4 memory resting and neutrophils in GC. This might provide a potential target for the future clinical immunotherapy strategy in GC patients.

However, this study has some limitations. Firstly, this is a retrospective study, and selection bias cannot be avoided. Prospective clinical studies in subsequent studies may help to further determine the accuracy of the nomogram model. Secondly, although two sets of data from TCGA and GEO databases for analysis and validation were used, further clinical data from other databases and even in several hospitals is needed to verify the accuracy of the results. Thirdly, we hypothesize that FTO and ALKBH5 serve as “m6A erasers” to regulate gastric cancer. However, this cannot be analyzed only *in silico*. Their potential functions and effects on immune microenvironment of tumors also should be verified in both *in vitro* and *in vivo* experiments. Fourthly, although FTO and ALKBH5 are likely to promote tumors, they also have a lot of irreplaceable functions, including correct splicing of mRNAs, cell cycle and mitosis checkpoint regulation, and thus depletion of these two genes could cause chromosome instability (https://www.pnas.org/content/115/2/E325 and https://pubmed.ncbi.nlm.nih.gov/30719031/). As a result, FTO and ALKBH5 are not likely to become ideal therapy targets. Fortunately, m6A “eraser” related risk genes may be potential alternatives.

In conclusion, our results suggest that the expression of m6A “eraser” related genes is associated with GC prognosis, and thus a nomogram model for predicting the OS was constructed. Also m6A “eraser” could play a potential role in regulating the occurrence and development of GC. In addition, m6A “eraser” led to some immune cell changes in GC patients, which might act as a potential target for immunotherapy of GC.

## Data Availability Statement

The datasets presented in this study can be found in online repositories. The names of the repository/repositories and accession number(s) can be found in the article/**Supplementary Material**.

## Author Contributions

JY and JL designed the study. XX, EZ, and JZ drafted the manuscript. XX, EZ, JZ, CZ, and YZ collected and performed all data analysis. All authors contributed to the article and approved the submitted version.

## Funding

This study was funded by the Grant of Clinical Research Promotion Program of Shanghai Ninth People’s Hospital, School of Medical, Shanghai Jiao Tong University (JYLJ201822).

## Conflict of Interest

The authors declare that the research was conducted in the absence of any commercial or financial relationships that could be construed as a potential conflict of interest.

## References

[1] Karimi P Islami F Anandasabapathy S Freedman ND Kamangar F. Gastric cancer: descriptive epidemiology, risk factors, screening, and prevention. Cancer Epidemiol Biomarkers Prev (2014) 23(5):700–13. 10.1158/1055-9965.Epi-13-1057 PMC4019373 24618998

[2] Bray F Ren JS Masuyer E Ferlay J. Global estimates of cancer prevalence for 27 sites in the adult population in 2008. Int J Cancer (2013) 132(5):1133–45. 10.1002/ijc.27711 22752881

[3] Soerjomataram I Lortet-Tieulent J Parkin DM Ferlay J Mathers C Forman D. Global burden of cancer in 2008: a systematic analysis of disability-adjusted life-years in 12 world regions. Lancet (London England) (2012) 380(9856):1840–50. 10.1016/s0140-6736(12)60919-2 23079588

[4] Zhao BS Roundtree IA He C. Post-transcriptional gene regulation by mRNA modifications. Nat Rev Mol Cell Biol (2017) 18(1):31–42. 10.1038/nrm.2016.132 27808276 PMC5167638

[5] Dai D Wang H Zhu L Jin H Wang X. N6-methyladenosine links RNA metabolism to cancer progression. Cell Death Dis (2018) 9(2):124. 10.1038/s41419-017-0129-x 29374143 PMC5833385

[6] Zaccara S Ries RJ Jaffrey SR. Reading, writing and erasing mRNA methylation. Nat Rev Mol Cell Biol (2019) 20(10):608–24. 10.1038/s41580-019-0168-5 31520073

[7] Yue B Song C Yang L Cui R Cheng X Zhang Z. METTL3-mediated N6-methyladenosine modification is critical for epithelial-mesenchymal transition and metastasis of gastric cancer. Mol Cancer (2019) 18(1):142. 10.1186/s12943-019-1065-4 31607270 PMC6790244

[8] Zheng G Dahl JA Niu Y Fedorcsak P Huang CM Li CJ. ALKBH5 is a mammalian RNA demethylase that impacts RNA metabolism and mouse fertility. Mol Cell (2013) 49(1):18–29. 10.1016/j.molcel.2012.10.015 23177736 PMC3646334

[9] Fedeles BI Singh V Delaney JC Li D Essigmann JM. The AlkB Family of Fe(II)/α-Ketoglutarate-dependent Dioxygenases: Repairing Nucleic Acid Alkylation Damage and Beyond. J Biol Chem (2015) 290(34):20734–42. 10.1074/jbc.R115.656462 PMC4543635 26152727

[10] Zhang S Zhao BS Zhou A Lin K Zheng S Lu Z. m(6)A Demethylase ALKBH5 Maintains Tumorigenicity of Glioblastoma Stem-like Cells by Sustaining FOXM1 Expression and Cell Proliferation Program. Cancer Cell (2017) 31(4):591–606.e6. 10.1016/j.ccell.2017.02.013 28344040 PMC5427719

[11] Li J Han Y Zhang H Qian Z Jia W Gao Y. The m6A demethylase FTO promotes the growth of lung cancer cells by regulating the m6A level of USP7 mRNA. Biochem Biophys Res Commun (2019) 512(3):479–85. 10.1016/j.bbrc.2019.03.093 30905413

[12] Zou D Dong L Li C Yin Z Rao S Zhou Q. The m(6)A eraser FTO facilitates proliferation and migration of human cervical cancer cells. Cancer Cell Int (2019) 19:321. 10.1186/s12935-019-1045-1 PMC6888952 31827395

[13] Su Y Huang J Hu J. m(6)A RNA Methylation Regulators Contribute to Malignant Progression and Have Clinical Prognostic Impact in Gastric Cancer. Front Oncol (2019) 9:1038. 10.3389/fonc.2019.01038 31681576 PMC6813557

[14] Irizarry RA Hobbs B Collin F Beazer-Barclay YD Antonellis KJ Scherf U. Exploration, normalization, and summaries of high density oligonucleotide array probe level data. Biostatistics (2003) 4(2):249–64. 10.1093/biostatistics/4.2.249 12925520

[15] Li W Li K Zhao L Zou H. Bioinformatics analysis reveals disturbance mechanism of MAPK signaling pathway and cell cycle in Glioblastoma multiforme. Gene (2014) 547(2):346–50. 10.1016/j.gene.2014.06.042 24967941

[16] Garcia-Laencina PJ Abreu PH Abreu MH Afonoso N. Missing data imputation on the 5-year survival prediction of breast cancer patients with unknown discrete values. Comput Biol Med (2015) 59:125–33. 10.1016/j.compbiomed.2015.02.006 25725446

[17] Pan YB Zhu Y Zhang QW Zhang CH Shao A Zhang J. Prognostic and Predictive Value of a Long Non-coding RNA Signature in Glioma: A lncRNA Expression Analysis. Front Oncol (2020) 10:1057. 10.3389/fonc.2020.01057 32793467 PMC7394186

[18] Vickers AJ Cronin AM Elkin EB Gonen M. Extensions to decision curve analysis, a novel method for evaluating diagnostic tests, prediction models and molecular markers. BMC Med Inform Decis Mak (2008) 8:53. 10.1186/1472-6947-8-53 19036144 PMC2611975

[19] Zhang C Zhang M Ge S Huang W Lin X Gao J. Reduced m6A modification predicts malignant phenotypes and augmented Wnt/PI3K-Akt signaling in gastric cancer. Cancer Med (2019) 8(10):4766–81. 10.1002/cam4.2360 PMC6712480 31243897

[20] Chen G Cao Y Zhang L Ma H Shen C Zhao J. Analysis of long non-coding RNA expression profiles identifies novel lncRNA biomarkers in the tumorigenesis and malignant progression of gliomas. Oncotarget (2017) 8(40):67744–53. 10.18632/oncotarget.18832 PMC5620208 28978068

[21] Wang W Yang F Zhang L Chen J Zhao Z Wang H. LncRNA profile study reveals four-lncRNA signature associated with the prognosis of patients with anaplastic gliomas. Oncotarget (2016) 7(47):77225–36. 10.18632/oncotarget.12624 PMC5363582 27764782

[22] Zhang J Guo S Piao HY Wang Y Wu Y Meng XY. ALKBH5 promotes invasion and metastasis of gastric cancer by decreasing methylation of the lncRNA NEAT1. J Physiol Biochem (2019) 75(3):379–89. 10.1007/s13105-019-00690-8 PMC6728298 31290116

[23] Zhang B Wu Q Li B Wang D Wang L Zhou YL. m(6)A regulator-mediated methylation modification patterns and tumor microenvironment infiltration characterization in gastric cancer. Mol Cancer (2020) 19(1):53. 10.1186/s12943-020-01170-0 32164750 PMC7066851

[24] Heuberger J Birchmeier W. Interplay of cadherin-mediated cell adhesion and canonical Wnt signaling. Cold Spring Harb Perspect Biol (2010) 2(2):a002915. 10.1101/cshperspect.a002915 20182623 PMC2828280

[25] Clements WM Wang J Sarnaik A Kim OJ MacDonald J Fenoglio-Preiser C. beta-Catenin mutation is a frequent cause of Wnt pathway activation in gastric cancer. Cancer Res (2002) 62(12):3503–6. 12067995

[26] Ooi CH Ivanova T Wu J Lee M Tan IB Tao J. Oncogenic pathway combinations predict clinical prognosis in gastric cancer. PLoS Genet (2009) 5(10):e1000676. 10.1371/journal.pgen.1000676 19798449 PMC2748685

[27] Sammarco G Varricchi G Ferraro V Ammendola M De Fazio M Altomare DF. Mast Cells, Angiogenesis and Lymphangiogenesis in Human Gastric Cancer. Int J Mol Sci (2019) 20(9):2106. 10.3390/ijms20092106 PMC6540185 31035644

[28] Chen J Chen JG Sun B Wu JH Du CY. Integrative analysis of immune microenvironment-related CeRNA regulatory axis in gastric cancer. Math Biosci Eng (2020) 17(4):3953–71. 10.3934/mbe.2020219 32987562

[29] Wang Y Zhai J Zhang T Han S Zhang Y Yao X. Tumor-Associated Neutrophils Can Predict Lymph Node Metastasis in Early Gastric Cancer. Front Oncol (2020) 10:570113. 10.3389/fonc.2020.570113 33072602 PMC7537418

