# Supplementary material for: Prognostic and Predictive Value of m6A “Eraser” Related Gene Signature in Gastric Cancer
Source: Front Oncol. 2021 Feb 26;11:631803. doi: 10.3389/fonc.2021.631803 (PMC7952866; doi:10.3389/fonc.2021.631803)
Supplement: Supplementary file 1 [file DataSheet_1.docx]

Supplementary Material

# Supplementary Figures and Tables

## Supplementary Figures


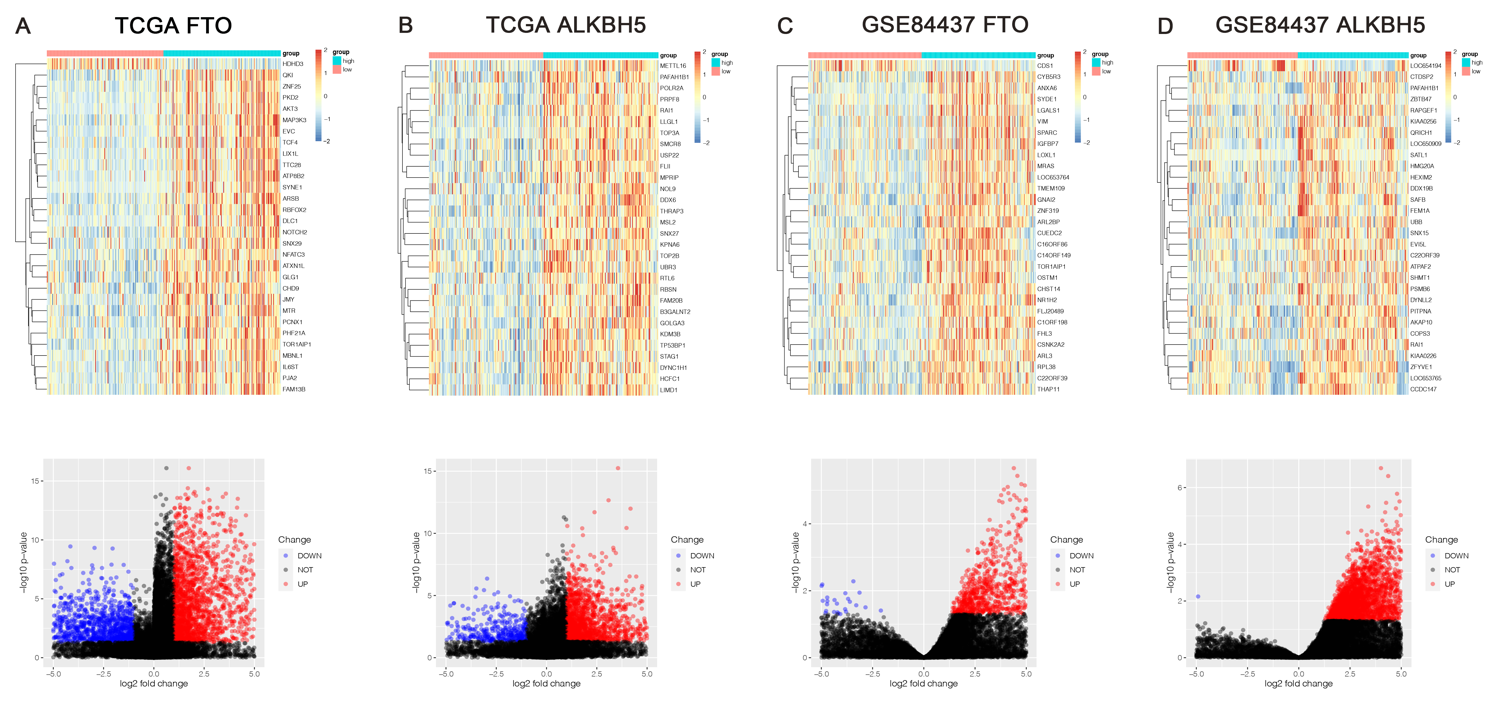


**Supplementary Figure 1.** The differential expression genes analysis of m6A “eraser” in gastric cancer. (A-D) The differences in the expression of top 30 differentially expressed genes between low and high FTO and ALKBH5 expression groups. Red and blue represents the relatively high or low expression, respectively; the volcano showed differentially expressed genes between low and high FTO and ALKBH5 expression groups.


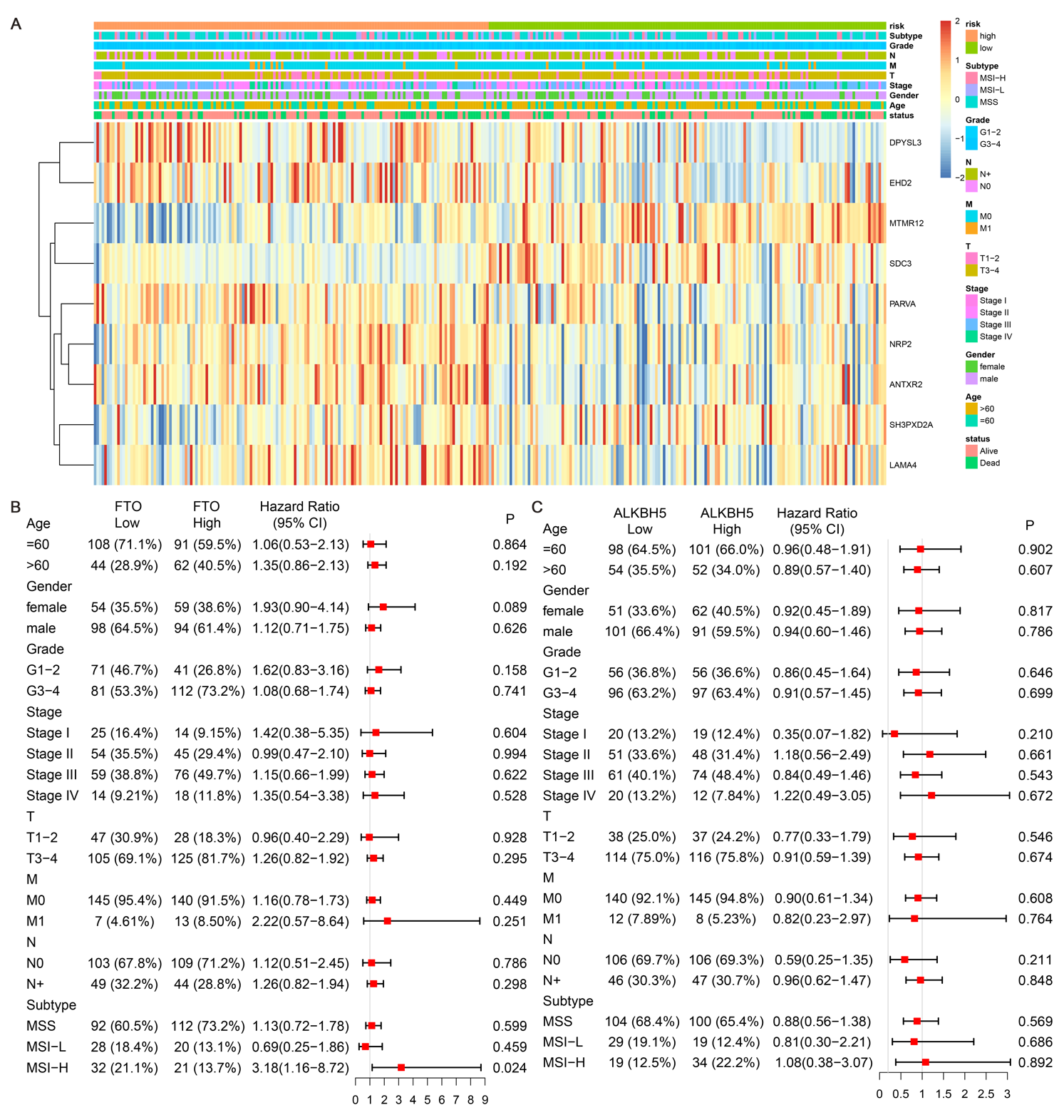


**Supplementary Figure 2.** The expression of 9 m6A “eraser” related risk genes with different clinicopathological characteristics and prognosis analysis in subtypes of each baseline clinicopathological characteristics group. (A) The heatmap showed the expression levels of the 9 m6A “eraser” related risk genes in low and high gastric cancer patients. The distribution of clinicopathological characteristics was compared between low and high groups. The prognosis analysis of FTO low and high groups (B) and ALKBH5 low and high groups (C) in subtypes of each baseline clinicopathological characteristics group was performed in TCGA datasets. The hazard ratios (HR) and 95% confidence intervals (CI) were calculated by univariate Cox regression.


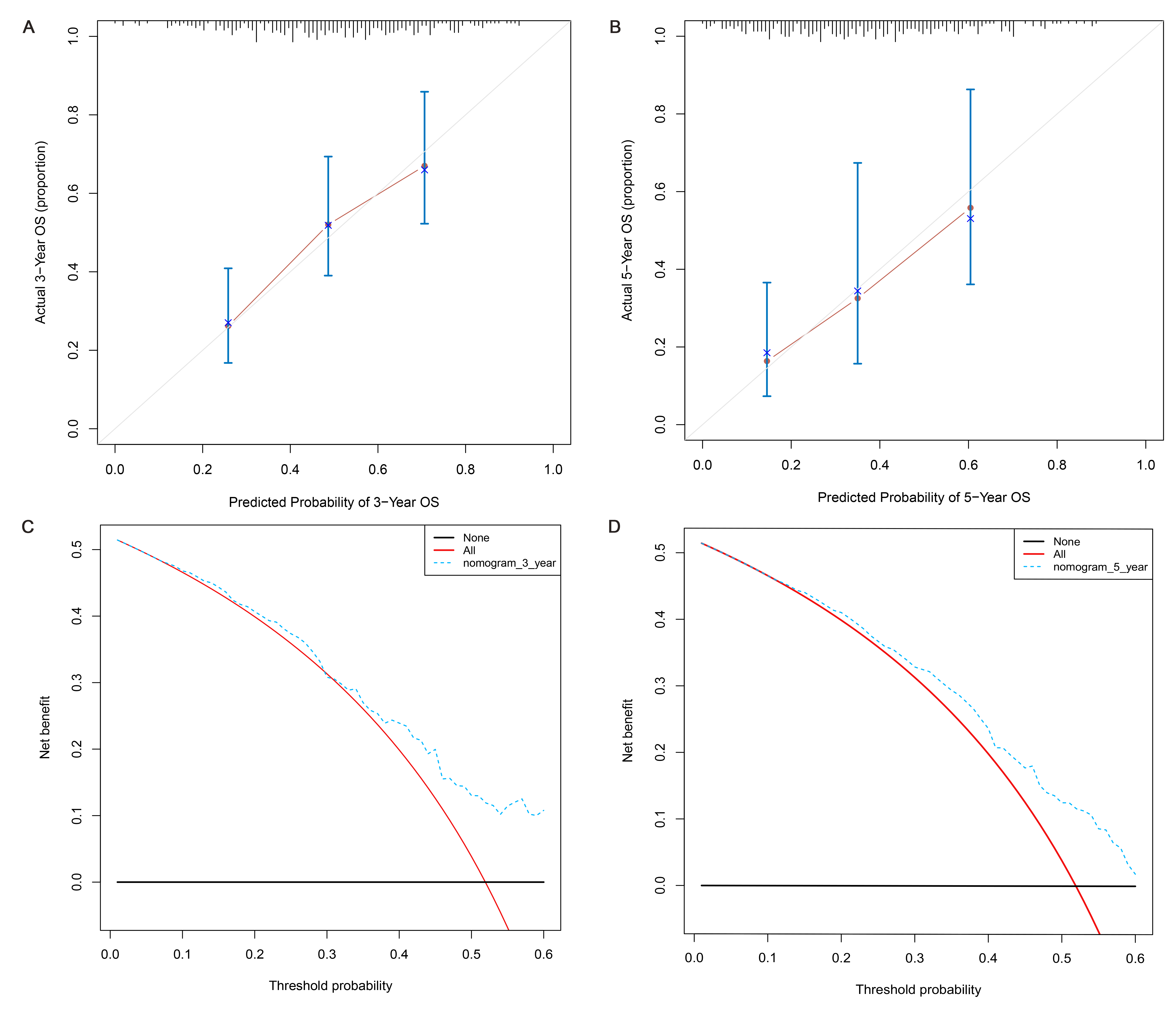


**Supplementary Figure 3.** The accuracy and clinical utility of nomogram prediction of 3-year and 5-year OS probability in gastric cancer. The calibration curve of the model was based on the consistency between predicted and observed 3- (A) and 5-year (B) outcomes in the TCGA dataset. Close-ended vertical lines represent 95% CIs. The x-axis represents the predicted OS probability and the y-axis represents the actual OS. The 45-degree line represents the perfect prediction. (C, D) Decision curve analysis (DCA) was used to assess the clinical utility of the nomogram. The x-axis represents the percentage of threshold probability and the y-axis represents the net benefit.


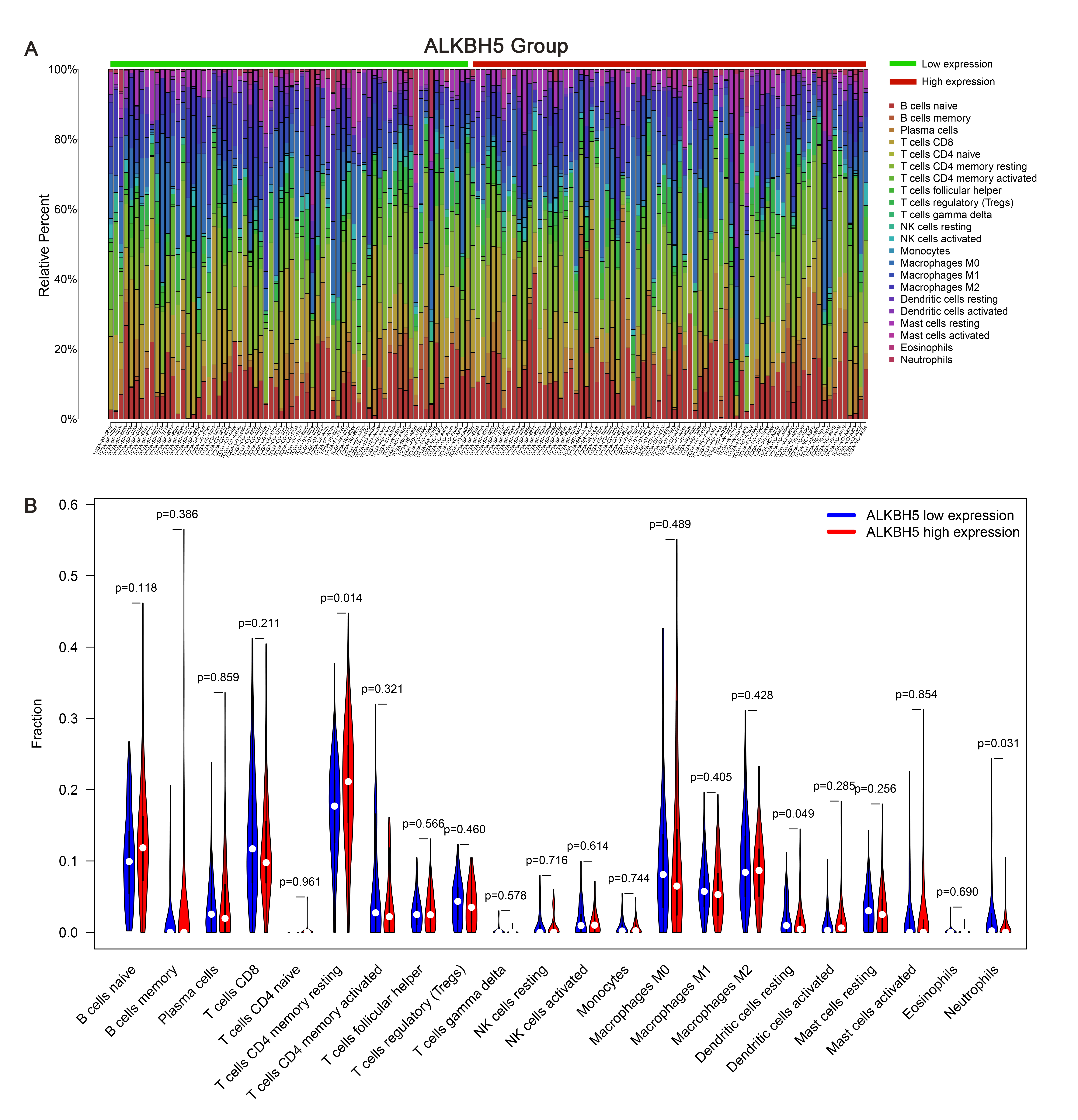


**Supplementary Figure 4.** The effect of ALKBH5 expression level on gastric cancer immune cells. (A) The proportion of immune cell subsets in the ALKBH5 high expression group and low expression group were analyzed in the TCGA dataset. (B) The violin map showed statistical differences between the immune cells of different ALKBH5 expression groups.

## Supplementary Tables

| **Gene** | **Ensemble ID** | **Description** | **Coefficient** |
| --- | --- | --- | --- |
| MTMR12 | ENSG00000150712 | Myotubularin-related protein 12 | -2.47335 |
| SDC3 | ENSG00000162512 | Syndecan-3 | -2.37796 |
| DPYSL3 | ENSG00000113657 | Dihydropyrimidinase-related protein 3 | 0.074203 |
| NRP2 | ENSG00000118257 | Neuropilin-2 | 3.74863 |
| ANTXR2 | ENSG00000163297 | Anthrax toxin receptor 2 | 0.455573 |
| EHD2 | ENSG00000024422 | EH domain-containing protein 2 | 0.463306 |
| SH3PXD2A | ENSG00000107957 | SH3 and PX domain-containing protein 2A | -2.71575 |
| LAMA4 | ENSG00000112769 | Laminin subunit alpha-4 | 0.626251 |
| PARVA | ENSG00000197702 | Alpha-parvin | 2.223197 |
| MTMR12 | ENSG00000150712 | Myotubularin-related protein 12 | -2.47335 |

**Supplementary Table 1.** Detailed description of m6A “eraser” related risk genes.
